# Exploring the Co-occurrence of Manual Verbs and Actions in Early Mother-Child Communication

**DOI:** 10.3389/fpsyg.2020.596080

**Published:** 2020-11-10

**Authors:** María José Rodrigo, Mercedes Muñetón-Ayala, Manuel de Vega

**Affiliations:** ^1^Facultad de Psicología, Universidad de La Laguna, San Cristóbal de La Laguna, Spain; ^2^Facultad de Comunicaciones, Universidad de Antioquia, Medellín, Colombia; ^3^Instituto Universitario de Neurociencias, Universidad de La Laguna, San Cristóbal de La Laguna, Spain

**Keywords:** verb-action co-occurrence, temporal synchrony, social interaction, embodied meaning, multisensory communication

## Abstract

The embodiment approach has shown that motor neural networks are involved in the processing of action verbs. There is developmental evidence that embodied effects on verb processing are already present in early years. Yet, the ontogenetic origin of this motor reuse in action verbs remains unknown. This longitudinal study investigates the co-occurrence of manual verbs and actions during mother-child daily routines (free play, bathing, and dining) when children were 1 to 2 (Group 1) and 2 to 3 (Group 2) years old. Eight mother-child dyads were video-recorded in 3-month intervals across 12 months (27 recording hours), and the timing of verbs and manual actions (21,876 entries) were coded by independent observers. Results showed that the probability of matched verb-action co-occurrences were much higher (0.80 and 0.77) than that of random co-occurrences (0.13 and 0.15) for Group 1 and Group 2, respectively. The distributions of the verb-action temporal intervals in both groups were quite symmetrical and skewed with the peak corresponding to both 0.00 s synchronic intervals (8% of the cases) and the shortest +5 s interval (40% of the cases). Mother-led instances occurred in both groups whereas child-led instances were restricted to Group 2. Mothers pragmatically aligned their verbal productions, since they repeatedly used (74%) those verbs they shared with their children’s repertoire (31%). In conclusion, the early multisensory communicative and manipulative scene affords grounding of verb meanings on the ongoing actions, facilitating verb-action pairing in the realm of social interactions, providing a new dimension to the prevailing solipsistic approach to embodiment.

## Introduction

A basic idea in the embodiment approach is that meaning is grounded on perceptual and motor processes (Glenberg, 1997; Barsalou, 1999; Pulvermüller, 1999; de Vega, 2008). For instance, reading or listening to the verb “to hammer” would briefly induce brain activations in the motor cortex, which partially overlap the networks involved in the real action of hammering. Beyond this intuitive idea, a large body of neuroimaging studies has reported activations in the motor and premotor cortex during the processing of action verbs (Pulvermüller, 2005). Convergently, neuropsychological evidence indicates that Parkinson’s disease is associated not only with movement dysfunctions, but also with selective difficulty to produce action verbs (Boulenger et al., 2006; Herrera and Cuetos, 2012; García and Ibáñez, 2018). Analyses of brain dynamics by means of EEG or MEG have also found that action verbs, compared to non-action verbs, modulate electrophysiological signatures associated with neural motor activity, such as desynchronizing the mu and beta rhythms (van Elk et al., 2010; Moreno et al., 2015; Klepp et al., 2019). Finally, single-pulse transcranial magnetic stimulation (TMS) applied over the hand primary motor cortex combined with the measure of motor-evoked potentials in a representative hand muscle demonstrated that processing manual verbs modulates the corticospinal excitability (Oliveri et al., 2004; Buccino et al., 2005; Papeo et al., 2009).

The above evidence comes from adults who have a well-established embodied semantic system. Yet, developmental studies have also shown that embodied effects on verb processing are already strikingly present in childhood (Wellsby and Pexman, 2014; Loeffler et al., 2016). For instance, a neuroimaging study reported that in 4- and 5-year-old children, motor areas of the brain were activated when they listened to verbs, but not when they listened to adjectives (James and Maouene, 2009). In another neuroimaging study children aged 5–7 were exposed to novel verbs while they actively manipulated the objects referred to or while they watched an experimenter interact with the objects, showing that the motor system was recruited while listening to the novel verbs only after learning involves self-generated interactions with objects (James and Swain, 2011). Even toddlers at 24 months of age, after listening to a familiar action verb, were able to predict the upcoming action in a video, indicated by their anticipatory eye movements toward the object referred to (Gampe and Daum, 2014). In an EEG study, toddlers between 18 and 27 months of age who heard action verbs and watched action video clips showed a significant suppression of the mu rhythm, a neural signature of motor processes, during both the processing of action verbs and the action videos, but not during the processing of pseudoverbs (Antognini and Daum, 2019). Altogether these findings suggest that: (a) there is a link between sensorimotor experience and language processing in the developing brain, as words that are associated with action elicit activation in the corresponding motor areas of the brain; (b) the re-activations of motor representations by listening to action verbs may require children to have actively interacted with objects; and (c) the sensorimotor system is already involved in the processing of action verbs at the beginning of verb acquisition.

While the previous studies address important issues, the origin of language embodiment is a question that remains unanswered. Based on the early presence of embodied effects, especially for action verbs, it is reasonable to propose that embodiment may be a consequence of the history of co-activation of linguistic and motor areas in the individual experience (Pulvermüller, 2008; Zwaan, 2008; Glenberg and Gallese, 2012). In turn, the history of co-activations presumably requires the presence of verb-action co-occurrences that can be traced back to the early infant’s communicative environment. Thus, a verb like “give” would acquire its grounded meaning in infancy from its repeated association with the actions of giving or receiving objects in interactions with parents/caregivers. To our knowledge, a systematic search for the timing and structure of verb-action co-occurrences in the naturalistic communicative environment accompanying the emergence of manual verbs has not been addressed. The present study tried to fill this gap by longitudinally exploring infants’ early communicative scenarios with their mothers to find out whether manual verb-action co-occurrences exist, and if so, in what ways and how they develop.

The logical precondition for the existence of verb-action co-occurrences is the presence of manual verbs and actions in the child’s early repertoire of linguistic and motor competences. Research has shown that during the first year (from 7 to 12 months of age) infants learn to manipulate objects, being able first to displace objects, then to separate them and finally to make constructions involving an assembly of several objects (e.g., Lifter and Bloom, 1989). At 13–15 months, infants have non-verbal categories of events and actions presented in the social scenario (Konishi et al., 2016), and by the end of the first year, they are able to understand many verbs, referring to trajectories of moving objects (e.g., “fall”), the outcomes of causal sequences (e.g., “open”), intentional actions (e.g., “get”), transactions (e.g., “give” and “take”), etc. (Behne et al., 2005). Yet, the first words produced by children include very few verbs, although they may refer indirectly to actions using other types of words (e.g., “up” to request getting up) (Huttenlocher et al., 1983; Tomasello, 1992). Only by the second year do children spontaneously produce action verbs (e.g., “give”), not only in terms of physical motions (“fall down”) but also in terms of underlying relations and goals (“gonna” or “find”) (Gleitman et al., 2005; Waxman and Lidz, 2006). Interestingly, longitudinal evidence demonstrates relationships between motor and language development since the increased possibilities for walking, exploring objects, and spatial layouts provide the cognitive basis for language learning (Iverson, 2010; Walle and Campos, 2014; Oudgenoeg-Paz et al., 2016). The correlational evidence supports the idea that the attainment of linguistic skills is grounded in specific sensorimotor experiences, which is a basic tenet of the embodied approach.

The developmental evidence also demonstrates a gap of around 1 year between the beginning of action performance and the production of the corresponding verbs, which may be due to difficulties in the word-referent pairing in real life contexts involving agents producing many words targeted at a variety of potential referents (Gentner, 2006; Poulin-Dubois and Forbes, 2006). However, to perform this pairing task, infants are not passively exposed to a chaotic input. They interact with their caregivers while they constantly deal with a wealth of multisensory, temporally organized information involving words, referents, and actions. According to the “intersensory redundancy hypothesis” the multisensory early communicative scenario is full of correlated visuo-spatial, motoric, and auditory information from which infants may establish patterns corresponding to relevant aspects of their environment which may receive selective attention (Bahrick and Lickliter, 2000; Reynolds et al., 2014). This hypothesis emphasizes the presentation of the same information spatially coordinated and temporally synchronous across two or more sensory modalities, which is only possible for amodal properties that are not specific to a single sense modality (e.g., shape, rhythm, duration, intensity). Infants seem to be prepared to benefit from multisensory and temporally synchronous presentation (Gogate and Hollich, 2010). The early emergence of multisensory integration capacity is supported by electrophysiological evidence (ERP) of enhanced neural responsiveness to synchronous compared to asynchronous audiovisual stimulation in 5-month-old infants (Reynolds et al., 2014). It is important to emphasize that the cross-modal input in communicative settings involves the infants’ selection of the relevant visual scene, thanks to their growing mobility, which allows them to interact with objects in play with their parents while hearing their verbalizations, supporting the developmental and social constraints that enable human infants to learn language from this complex data (Yu and Smith, 2012, 2016).

The acquisition of action verbs may also benefit from this communicative scenario, since the performance of perceptually salient actions in the temporal vicinity is one possible way to facilitate the verb-referent pairing, thereby creating a rich time-locked set of experiences (Hollich et al., 2000; Katerelos et al., 2011). Thus, for 21-month-old children, mapping between verbs and perceptually salient actions is easier than mapping between verbs and uninteresting actions (Brandone et al., 2007). Also, the repeated occurrence of verbs and their corresponding concrete actions facilitates the acquisition of novel verbs in 24- or 30-month-old infants (Childers and Tomasello, 2006; Poulin-Dubois and Forbes, 2006). Moreover, most of a child’s (age 24–26 months) utterances with verbs in typical mother-child contexts refer to their own actions (Huttenlocher et al., 1983), although in wider contexts (Naigles et al., 2009) or when requested (Childers and Tomasello, 2006), they were also able to describe others’ actions. The input of mothers performing actions in synchrony with their action verbs to 6-month-old infants predicted the number of spoken verbs at 24 months (Nomikou et al., 2017). A related phenomenon in verb learning is that in mother-child speech, a single verb is typically found in many phrasal patterns involving different objects, facilitating a fast mapping between a phrasal form and its meaning (Casenhiser and Goldberg, 2005). It still remains to be explored to what extent in the natural communicative environment there are verb-action occurrences either coincident or closer in time involving different objects/referents, in which the child, the mother or both might be involved.

The general objective of the present study was to examine longitudinally the temporal correspondence between the production of manual verbs and the performance of manual actions by the child and by the mother in their communication during daily routines over the second and third years of life. Knowing this verb-action temporal correspondence may be crucial to helping us understand the conditions in which manual verb meaning is acquired. We focused on the distribution of temporally synchronic or delayed verb-action instances recorded during the spontaneous exchanges between mother and child. In particular, we examined manual verbs (herein verbs) that refer to concrete goal-directed actions performed with the hands (e.g., catching a ball) that appear very frequently in infant and adult speech (Tomasello, 1992; Hirsh-Pasek and Golinkoff, 2006), produced in a temporally coordinated manner with manual actions directed to objects, like catching something (herein actions), including either one (mother or child) or two agents (mother and child). Compared to postural verbs involving bodily changes, manipulative actions are well researched in the embodiment literature, they are transitive (have a referent object to explore the referential space), and they are represented with more types in the early repertoire of Spanish and English verbs.

We started the search for matched verb-action (the verb corresponds to the action) co-occurrences at the end of the first year (12 months) and finished at the end of the third year (36 months). As mentioned above, at 12 months infants skillfully manipulate objects (Lifter and Bloom, 1989) and understand adults’ utterances with action verbs (Behne et al., 2005), but they do not produce action verbs until the second year (Gleitman et al., 2005). This comprehension-production gap in the first year of life makes especially relevant the co-occurrence of mothers’ verbs and infants’/mothers’ actions that may facilitate the verb-referent pairing in absence of infants’ verb production. We opted for naturalistic home routines observed over lengthier periods and at several developmental time points, which may reveal an ecologically more valid and varied picture of child-mother communicative environments than shorter and structured lab situations (Tamis-LeMonda et al., 2017). The temporal intervals between verb production and action performance were computed from the video-recorded flow of mother-child exchanges, accurately preserving the onset times of verb and action data. In this way, we built up a sizable corpus of child and mother verb and action production, comprising 21,876 entries (3,749 verbs and 18,127 actions). In addition to the onset time, several parameters were recorded for each entry: who is speaking/acting, what he/she is saying (verb) or doing (action), and what was the object manipulated and/or referred to by the verb.

Two specific objectives were addressed. First, to examine whether matched verbs and actions co-occurred beyond chance level, to determine the minimum verb-action interval that comprises the largest frequency of matched instances and to describe the agents involved. Among those instances, we further examined whether there were age months/group differences in both the temporal distribution of matched verb-action instances and the type of agent involved (child, mother, or both). We expected that the probability of matched verb-action instances would be higher than the probability of random co-occurrence of verbs and actions. We also expected that the majority of instances would correspond to delayed verb-action instances, being synchronic instances a small proportion in both age groups. With respect to the agents involved, we predicted that both mothers and infants would perform manual actions in both age groups and in all sessions. However, the production of verbs would be almost exclusive to mothers in the second year (12–24 months), meaning that most matched verb-action instances would be mother-led instances, namely, “Mother verb–Child action” and “Mother verb–Mother action.” Only in the third year (24–36 months) would children consolidate the production of verbs, and child-led instances would also be developed: “Child verb–Child action” and “Child verb–Mother action.” In sum, we predicted that matched verb-action instances would occur beyond chance level and in close temporal proximity, and that mother-led matched instances would dominate interchanges in the first age group (also across sessions) and both mother-led and child-led instances would be consolidated in the second age group (also across sessions).

The second objective was to examine how constrained the child and the mother verbs’ referential spaces are, and the degree of type and token redundancy in the shared repertoire of verbs within matched instances. Referential space is indexed by the number of different objects referred to, resulting in a continuum of lower to higher verb specificity (Maouene et al., 2011). For instance, verbs such as “make,” “take,” or “bring” are quite general and label a wide range of motor actions addressed to a great variety of objects, whereas verbs such as “carry,” “pour,” or “paint” seem to refer to relatively specific motor actions, involving a low variety of objects. Children appear to be learning both general and specific verbs at the same time (Naigles et al., 2009; Childers, 2009). Those verbs that can be considered general with a high proportion of associated objects may facilitate a fast mapping with their meaning (Casenhiser and Goldberg, 2005). As for redundancy, a high overlapping of types and tokens would mean that the child is exposed to the same verbs under different conditions (agent and situation), which may facilitate their generalizability (Poulin-Dubois and Forbes, 2006). Therefore, we would tentatively expect the presence of maternal verb adaptations (general referential space and higher redundancy) in the constrained space of matched instances.

The two objectives are closely related, since they aim to reveal crucial features of the early communicative scene that form the basis of action verb meaning. The first emphasizes the situational parameters that facilitate the verb-action pairing (match, synchronicity, and intervening agents), whereas the second examines some semantic (referential field) and pragmatic properties (redundancy) that also may favor verb-action pairing.

## Materials and Methods

### Participants and Procedure

Over the course of 12 months, with an interval of 3 months (five sessions per dyad), the activity sequences of four 1-year-old Spanish children and their mothers (Group 1) and four 2-year-old Spanish children and their mothers (Group 2) were recorded. The mean age of the children in Group 1 was 1;4 (SD = 0.2) and the mean age of children in Group 2 was 2;3 (SD = 0.1) at the time of first observation session. Mothers with infants were recruited using flyers spread at the university centers. All children were first-born, typically developing infants, and all had mothers (mean age 29, range 26–34 years, for both groups) with a university education and medium to high socioeconomic status. Four children had mothers who worked outside their home, and four children had mothers working at home (half in each age group). This study was carried out in accordance with the recommendations of the Ethics Committee of the University of la Laguna, Spain. In accordance with the Declaration of Helsinki, a written informed consent was obtained from the mothers.

### Setup and Verb and Action Coding

**FIGURE 1:**
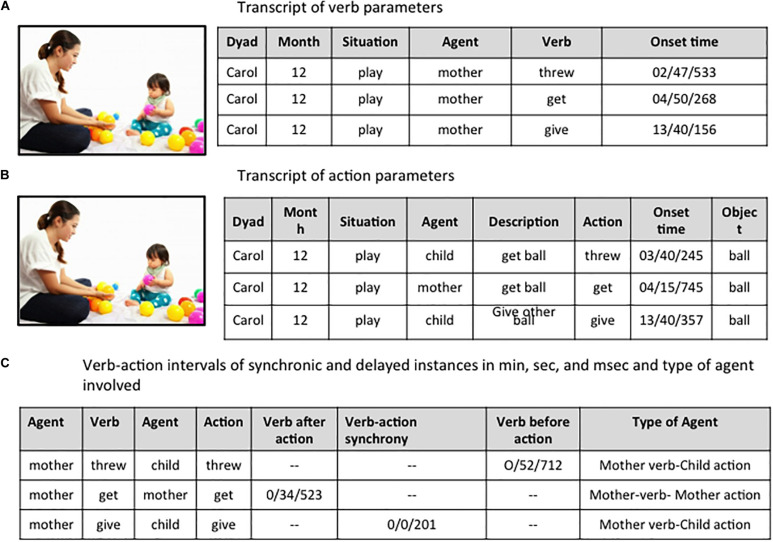
The matrix of observational data was created incrementally for each dyad, month, and situation following three steps: **(A)** Literal transcription of manual verbs and onset time from the videos; **(B)** Watching the muted videos to collect manual actions, onset time, and object involved; and **(C)** Temporal alignment of mother-child verbs and actions, to record onset of verb-action instances in synchronic and delayed instances and agents involved. Different observers were used at **(A)**, **(B)** and **(C)** phases. The image used to illustrate the coding procedure has been obtained from a public database freely available: https://es.dreamstime.com/imagen-de-archivo-el-beb%C3%A9-est%C3%A1-jugando-la-bola-con-su-madre-image32992441.

Video recordings were made of activity sequences during free play, followed by bathing and, finally, eating dinner. Observer arranged with the families to pay a visit to them during the regular evening sequence. Mothers were instructed to interact and play with their children as they normally would; meanwhile, the observer avoided interfering with mother-child interactions. The same observer recorded all sessions for each dyad and across dyads. A portable video camera MV3-01B-BL Mevo Start with three microphones for live events recorded the activity, and two MacBook Pro computers, connected to a recorded chronometer with an accuracy of milliseconds were used to code the data that was registered in a Microsoft Excel sheet. Figure 1 provides an outline of the coding procedure with an example (fictitious name and illustration) translated into English involving matched verb-action instances in which the verb corresponds to the action, which produced part of the data matrix for further analyses.

As for the coding procedure, different observers coded each stream of language data and action data independently, and the onset of each instance was noted in real-time with the recorded chronometer with an accuracy of milliseconds, for each dyad/session/situation. The manual verbs were recorded and literally transcribed even when they were poorly pronounced, their tense could not be determined (e.g., there was no temporal inflection) or their inflection was incorrect, to avoid underestimating the younger children’s verbal capabilities. Manual verbs were coded when the speaker referred to handling objects with instruments [“peina la muñeca” (“*comb the doll’s hair*”)] or without instruments [“coge el jabón” (“*pick up the soap*”)]. Manual actions involved the person’s use of his/her hands to perform a meaningful goal-directed action on an object with or without a tool (e.g., picking up the soap; giving a ball to the adult). A brief description of the action was also recorded. Incomplete muscular movements without a clear goal (open the mouth or lifting the arm) were not coded. Postural verbs and actions referred to body movements involving either a spatial displacement or a change of position (e.g., sitting, standing up, coming) were not considered. Inter-rater reliability of all the data was assessed with Cohen’s Kappa coefficient, 0–1, and was adequate (higher than 0.80): for verbs it was 0.89 (Group 1) and 0.88 (Group 2); and for actions it was 0.88 (Group 1) and 0.87 (Group 2).

Table 1 gives descriptive data about identification initials, age group, sex, number of sessions recorded, number of videotaped minutes, number of manual verbs produced, and number of actions performed by the child and by the mother. Total observation time was 27 h. Table 1 shows a massive presence of 18,127 actions (11.2 per minute) with respect to the verb production of 3,749 instances (2.3 per minute). Verb production was eight times larger in mothers with respect to child production, whereas action performance was just 1.3 times larger in mothers with respect to child performance.

**TABLE 1:** Comparative child and mother data on total manual verbs and actions.

**Child**	**Age group**	**Sex**	**Number of sessions**	**Total time**	**Child**		**Mother**	
					**Total verbs**	**Total actions**	**Total verbs**	**Total actions**
PA	1–2	F	5	179’	6	1047	260	1154
LA	1–2	F	5	186’	0	548	412	1418
JP	1–2	M	5	185’	1	592	282	777
CA	1–2	M	5	271’	3	663	452	1285
CR	2–3	F	5	141’	18	510	435	712
PC	2–3	M	5	221’	62	889	230	1423
PB	2–3	M	5	196’	124	1249	649	1425
CE	2–3	M	5	244’	199	2290	616	2145
Total			40	1623’	413	7788	3336	10,339

*Sessions and situations (play, bath, and dinner) are aggregated.*

### Coding of Verb-Action Instances

At phase (C) in Figure 1 the temporal alignment of the mother and child verbs and actions was performed by interweaving the verb and action segments in real-time and sequentially for each dyad, month, and situation. From this combined matrix two observers identified verb-action instances and calculated their interval times. Previous studies have shown that the Mother action may come either before or after the Mother verb in the input of 6-month-old infants (Nomikou et al., 2017). In our study, the following decisions were made: (a) to identify each verb token, recording its onset time; (b) to identify any matching action with the verb, within a temporal window extending from the onset of the previous verb to the onset of the following verb, recording the onset of the matching action; and (c) to calculate the actual verb-action interval time rather than establishing an arbitrary time window to analyze matching events; and (d) to record, for each matched verb-action instance, the manipulated object that was the referent target of the verb. We also counted mismatched verb-action instances in which the action does not correspond to the verb in the critical time window between the onset of the preceding and the following verb. Inter-rater reliabilities (Cohen’s Kappa coefficient, 0–1) with all instances were high: 0.95 for synchronic instances, 0.86 for displaced instances, and 0.85 for mismatched instances.

Categories and real examples of the verb-action instances translated into English are the following: (1) synchronic instances; for instance, the child tries to take off his shirt while he says “*take off*”; (2) delayed verb instances (verb following action); for instance, the child catches the soap and afterward the mother says: “*you caught the soap*”; (3) delayed action instances (verb preceding action); for instance, mother says: “*I am going to turn on the tap*” and afterward she turns on the tap; and (4) mismatched verb-action instances in which we identify two cases: those in which mothers’ descriptions, prompts and questions place the focus of the discourse on other actions that are not currently being undertaken (e.g., mother feeds her baby and says *“I am going to pick up daddy”*), and those cases in which the mothers’ descriptions, commands, or questions necessarily demand a delayed behavioral response far from the present routine (e.g., mothers gives the spoon to the baby and says *“after dinner you can bring me the doll”*).

### Plan of Analyses

For the first objective, namely, exploring the verb-action co-occurrences and their timing, we followed two steps. In step 1, we estimated the probability of random verb-action co-occurrences, multiplying the probabilities of the isolated constituents (verbs and actions) for each age group. Random occurrences of combined events correspond to the probability that any verb can be associated with any action in the whole temporal stream. Then, we estimated the probability of matched verb-action co-occurrences for each age group. Matched co-occurrence was computed as the probability that the target verbs were associated with the corresponding actions in the whole temporal stream. In spite of this strict criterion, we expected that the matched probability of co-occurrences would outnumber the random probability of occurrences of combined events.

In step 2, after inspecting the changes by sessions, we created an aggregated histogram with the distribution of matched verb-action instances across different temporal intervals within a broad time window of ±60 s from the verb onset. We aimed at finding the minimum verb-action interval, presumably in the range of few seconds, of the matched instances that comprises the largest frequency of instances for each age group. The synchronic verb-action occupied the central position in the *x*-axis. Progressively toward the left were those time intervals coded with a negative sign corresponding to the delayed verb instances, and progressively toward the right were those intervals coded with a positive sign corresponding to the delayed action instances. A synchronic instance is the one in which the temporal interval between the verb and the action is 0.00 s, once calculated the difference between verb and action onset times. A delayed instance is the one in which the temporal interval between the verb and the action is >0.00 s, once calculated the difference between verb and action onset times. The histograms were graphed for each age group (aggregating five sessions and three situations) and then by type of agent (aggregating four dyads) involved in each age group. The mean, mode, and median range of the distribution as well as its skewness and kurtosis were computed for each age group, as well as for the agents involved: Mother verb–Mother action, and Mother verb–Child action; and two led by the child: Child verb–Child action and Child verb–Mother action, are logically expected.

To explore the second objective, the amplitude of the verb referential space and their type and token redundancy in mothers and children, we focused on the verb production within the mother-led and child-led categories found. We first made a list of verbs shared by the mother and the child, collapsing age groups, with values of frequency and percentage, and the number of objects referred to per verb for each agent’s category. To estimate how constrained are the child and mother verbs’ referential spaces, we counted the number of different objects referred to by each verb across the instances in which that verb was produced in the mother and child data and divided by the verb frequency to make the results comparable among verbs. To compute the degree of type and token redundancy separately (in %), we computed the number of types and tokens in the verb repertoire shared by the mother and child against the total mother’s production in the mother-led verb-action categories.

## Results

Table 2 shows the child’s and the mother’s separate production and rate per minute of verbs and actions in each session. The pattern was similar across situations and data was aggregated. As expected, in Group 1 children’s verb production emerged late and at a slow pace at 21–24 months, whereas they performed manual actions from the first session at the rate of 3.5 per minute in Group 1 and 6.2 in Group 2. Children’s verb production in Group 2 was more robust, approaching one verb per minute at 30–36 months. As expected, mothers produced verbs steadily from the first session in Group 1 at a rate of more than one verb per minute, increasing the rate to almost 2.5 verbs per minute in Group 2. Mothers performed actions steadily from the first session in Group 1 and kept the production quite stable across sessions from almost 6 actions per minute to 7 actions per minute in Group 2.

**TABLE 2:** Number of occurrences and rate per minute (in parenthesis) of verbs and actions calculated separately by child and mother data, and by session.

**Session/months**	**Total time**	**Child**		**Mother**	
		**Total verbs**	**Total actions**	**Total verbs**	**Total actions**
12	114’	0	243 (2.13)	228 (2.0)	848 (7.44)
15	157’	0	615 (3.92)	185 (1.18)	687 (4.38)
18	163’	0	489 (3.0)	241 (1.48)	1011 (6.20)
21	206’	3 (0.01)	715 (3.47)	333 (1.62)	988 (4.80)
24	181’	7 (0.04)	788 (4.35)	419 (2.31)	1100 (6.08)
Total	821’	10 (0.01)	2850 (3.47)	1406 (1.71)	4634 (5.64)
24	131’	25 (0.19)	701 (5.35)	310 (2.37)	876 (6.69)
27	179’	30 (0.17)	747 (4.17)	402 (2.25)	1060(5,92)
30	174’	117 (0.67)	1553 (8.93)	380 (2.18)	1555 (8.94)
33	177’	129 (0.73)	1314 (7.42)	483 (2.73)	1564 (8.84)
36	141’	102 (0.72)	623 (4.42)	355 (2.52)	650 (4.61)
Total	802’	403 (0.5)	4938 (6.16)	1930 (2.41)	5705 (7.11)
Grand total	1623’	413 (0.25)	7788 (4.79)	3336 (2.05)	10,339(6.37)

*Participants and situations (play, bath, and dinner) are aggregated. Data from 36-month session are reduced due to the accidental erase of a video in one dyad (bath situation).*

### Probability of Random and Matched Verb-Action Co-occurrences

For the first objective, in Table 3 we compared the probability of random versus matched verb-action co-occurrences for each age group in the whole temporal stream, from a total number of 3,749 verbs (1,416 in Group 1 and 2,333 in Group 2) and 18,118 actions (7,484 in Group 1 and 10,643 in Group 2) in Table 2. The probability that any verb occurred with any action at random was similar and very low at both age groups (0.13 and 0.15). By contrast, within the restricted set of matched verb-action instances (1,134 in Group 1 and 1,794 in Group 2), the probability that a given verb co-occurred with the matched action was five to six times higher in each group (0.80 and 0.77). The probability of mismatched instances (282 in Group 1 and 539 in Group 2) had the complementary values (0.20 and 0.23). The percentage of mismatched instances corresponding to 12, 15, 18, 21, and 24 months were respectively: 7.8, 7.8, 14.2, 23, and 47.2% and restricted to the mother verb production showing substantial increases from age 18 months on. The percentage of mismatched instances corresponding to 24, 27, 30, 33, and 36 months were respectively: 13, 31.8, 21.6, 20.1, and 13.5% for the mother verb production with a more sustained use; and 1.3, 11.6, 23.4, 24.7, and 39% for the child verb production, showing an abrupt and sustained increase at age 30 months.

**TABLE 3:** Frequencies and probabilities of individual events (verbs and actions) and of combined events with random and matched verb-action co-occurrences by group.

	**Individual events**			**Combined events**		
	**Total event occurrences (verbs + actions)**	**Total verb occurrences**	**Total action occurrences**	**Random verb-action occurrence**	**Matched verb-action co-occurrence**	
	**Frequency/probability**	**Frequency/probability**	**Frequency/probability**	**Random probability**	**Frequency of matches/total verbs**	**Matched probability**
Group 1	8900/1.0	1416/0.16	7484/0.84	0.13	1134/1416	0.80
Group 2	12 976/1.0	2333/0.18	10 643/0.82	0.15	1794/2333	0.77

*Participants, sessions, and situations are aggregated. Random probability is the product of probabilities of verbs and actions.*

### Distribution of Matched Verb-Action Instances by Group and Agency

**FIGURE 2:**
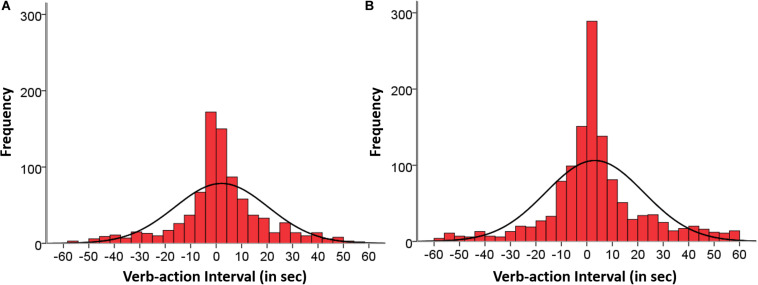
Temporal distributions of matched verb-action instances in **(A)** Group 1 (865 instances) and **(B)** Group 2 (1,281 instances), occurring within the span of ±60 s and aggregating participants, sessions, and situations.

**FIGURE 3:**
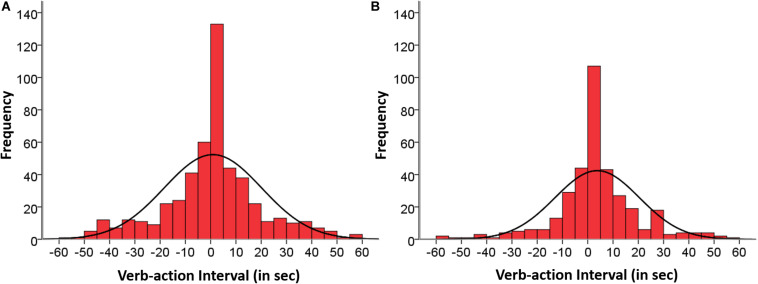
Group 1: Temporal distribution of matched verb-action instances corresponding to two mother-led categories: **(A)** Mother verb–Mother action (506 instances), and **(B)** Mother verb–Child action (355 instances), occurring within the span of ±60 s and aggregating participants, sessions, and situations.

**FIGURE 4:**
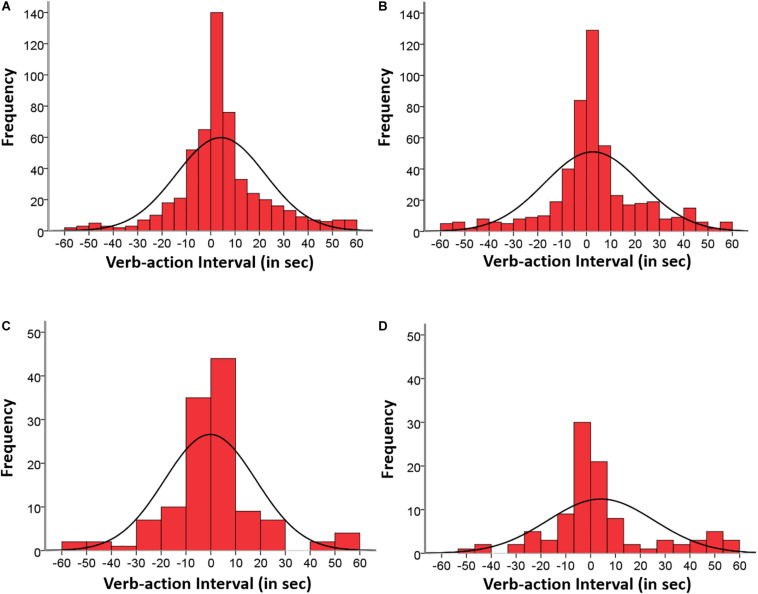
Group 2: Temporal distribution of matched verb-action instances corresponding to the four agent categories: **(A)** Mother verb–Mother action (549 instances), **(B)** Mother verb–Child action (509 instances), **(C)** Child verb–Child action (123 instances), and **(D)** Child verb–Mother action (100 instances), occurring within the span of ±60 s and aggregating participants, sessions, and situations.

Figures 2–4 depict the distributions of matched verb-action instances within the temporal window ±60 s from the verb onset by age groups and agents involved. This time window comprises 76.3% of the total co-occurrence in Group 1 (865 instances) and 71.4% of the total co-occurrence in Group 2 (1,281 instances). Table 4 reports descriptive parameters of these histograms: mean, SD, mode, skewness, and kurtosis. Figure 2 shows the histograms of matched verb-action instances corresponding to (A) Group 1 and (B) Group 2, and Table 4 includes the descriptive data. The distributions were approximately symmetrical, although with slightly more delayed action (53%) than delayed verb (37.8%) instances, and also highly leptokurtic for both groups, peaking around the mode = 0, corresponding to synchronicity. The synchronic verb-action interval of 0.00 s comprises 8.7 and 7% of the cases; ±2 s comprises 22 and 18.3% of the cases; ±5 s comprises 40% of the cases in both groups; ±10 s comprises 58 and 61.1%; and ±30 s 87 and 86% of the cases, respectively.

**TABLE 4:** Descriptive data for the temporal distributions of matched verb-action instances by group and agents.

**Figure**	**Group**	**Agents**	**Mean (SD)**	**Mode**	**Skewness (SE)**	**Kurtosis (SE)**
Figure 2	Group 1A	All	1.9 (18.3)	0	−0.01(0.08)	1.64 (0.16)
	Group 2B	All	3.2 (19.2)	0	0.14 (0.07)	1.67 (0.14)
Figure 3	Group 1A	Mother verb–Mother action	0.78 (19.3)	0	−0.01(0.11)	1.22 (0.22)
	Group 1B	Mother verb–Child action	3.48 (16.7)	0	0.08 (0.13)	2.54 (0.25)
Figure 4	Group 2A	Mother verb–Mother action	4.11 (18.3)	0	0.23 (0.10)	1.90 (0.21)
	Group 2B	Mother verb–Child action	2.82 (19.8)	0	−0.08(0.10)	1.47 (0.21)
	Group 2C	Child verb–Child action	−0.22(18.4)	0	0.32 (0.22)	2.72 (0.43)
	Group 2D	Child verb–Mother action	4.26 (21.4)	0	0.65(0.24	0.92 (0.47)

*Participants, sessions, and situations are aggregated. Skewness values between −0.5 and 0.5 indicate that the distribution is approximately symmetric; kurtosis values greater than +1 indicate that the distribution is too peaked.*

In Group 1 only categories led by the mother: “Mother verb–Mother action” (506 instances), and “Mother verb–Child action” (355 instances) comprised a substantive number of instances, whereas the other two categories led by the child (“Child verb–Child action,” and “Child verb–Mother action”) comprised two instances each only, reflecting the poor verbal performance in this age group. The percentage of “Mother verb–Mother action” instances corresponding to 12, 15, 18, 21, and 24 months were respectively: 20.9, 13.1, 22.3, 18.2, and 25.5% occurring from the beginning and sustained across age. The corresponding percentages of “Mother verb–Child action” instances were respectively: 11.3, 19.7, 23.4, 21.1, and 24.5% occurring from the beginning and sustained across age. Figure 3 shows the aggregated histograms of Group 1 that included the distribution of matched verb-action instances signaling the agents involved from the same data set, with descriptive data in Table 4. The distributions of both categories were symmetrical with an overall tendency of more delayed action (50.6%) than delayed verb (40.7%) instances, once the synchronic cases had been discounted. The synchronic verb-action instances (0 s) comprise 5 and 8.7% of the cases; instances in the ±2 s interval comprise 22.5 and 21.1%; instances in the ±5 s interval comprise 38.3 and 42% of the cases; in the ±10 s interval they comprise 55.1 and 63.1%; and in the ±30 s interval they are 84.8 and 91.2% of the cases, respectively.

In Group 2 the two mother-led categories and the two child-led categories comprised a substantial number of instances. The percentage of “Mother verb–Mother action” instances corresponding to 24, 27, 30, 33, and 36 months were respectively: 17.3, 18.4, 20, 26.6, and 17.7% [the decrease at 36 months in mother and child data was due to the accidental erase of a video in one dyad (bath situation)]. The corresponding percentages of “Mother verb–Child action” instances were respectively: 17.3, 15.1, 22.8, 29.1, and 15.7%; both occurring from the beginning and were sustained across age. Mother and child data from 36-month session are reduced due to the accidental erase of a video in one dyad (bath situation; see also note in Table 2). Figure 4 displays the aggregated histograms of Group 2 including the matched verb-action instances distributed by type of agent, with descriptive data in Table 4. The two mother-led categories are shown in the upper part (A) “Mother verb–Mother action” (549 instances), and (B) “Mother verb–Child action” (509 instances). The two distributions were approximately symmetrical with an overall tendency of more delayed action (55.9%) than delayed verb (43.9%) instances, and highly leptokurtic. The synchronic verb-action interval of 0 s comprises 7.8 and 5.5% of the cases; ±2 s comprises 18.3 and 17.7%; ±5 s comprises 40.1 and 42% of the cases; ±10 s comprises 60.8 and 60.7%; and ±30 s 87.8 and 84.9% of the cases, respectively.

The percentage of “Child verb–Child action” instances corresponding to 24, 27, 30, 33, and 36 months were respectively: 11.4, 4.9, 21.1, 41.5, and 21.1%. The corresponding percentages of “Child verb–Mother action” instances were respectively: 7, 4, 37, 38, and 14%, showing in both cases an abrupt increase at age 30 months. Figure 4 also displays at the bottom part the aggregated histograms of child-led categories: (C) “Child verb–Child action” (123 instances), and (D) “Child verb–Mother action” (100 instances), with descriptive data in Table 4. The distribution of the two categories was quite symmetrical in the first case (45% each half) and moderately skewed in the second case with an inverted tendency of more delayed verb (46.3%) than delayed action (43.1%) cases. The synchronic verb-action interval of 0 s comprises 9.7 and 7% of the cases; ±2 s comprises 21.1 and 22%; ±5 s comprises 42.3 and 43% of the cases; ±10 s comprises 65 and 63%; and ±30 s 91 and 82% of the cases.

### Referential Space and Redundancy in the Mother-Child Shared Verb

For the second objective, we identified first the repertoire of verbs shared by the mother and child in their productions of matched verb-action instances, and then we computed the number of different objects associated with each verb, as indicators of verb referential space (see Supplementary Table 1). In the two child-led categories (“Child verb–Child action” and “Child verb–Mother action”) the number of objects by verb frequency proportion (NObj/Vf) was quite high (0.54 and 0.66), showing that the majority of verbs (89%) can be considered general with a high proportion of associated objects (e.g., “catch”: 0.89; “have”: 0.75). There were also a few specific verbs, which involved a low proportion of associated objects (“throw”: 0.24; “break”: 0.25). For the same set of verbs in the two mother-led categories (“Mother verb–Mother action” and “Mother verb–Child action”) there was a greater mixture of general (59%) and specific verbs, since the NObj/Vf was lower (0.35 and 0.37), showing examples of general verbs (e.g., “cut”: 0.71; “break”: 0.69) as well as more specific verbs (e.g., “comb”: 0.18; “catch”: 0.25).

Table 5 indicates the type and token redundancy in the verb repertoire shared by the mother and child in mother-led categories (“Mother verb–Mother action” and “Mother verb–Child action”) compared to the total mother production (shared and non-shared verbs), showing a much lower overlap in types (31.6 and 31.2%) than in tokens (74.8 and 73%), respectively.

**TABLE 5:** Type and token redundancy (in %) in the mother-child shared repertoire (first column) against the total mother’s verb production (second column) in the mother-led verb-action categories of matching instances found within ±60 s interval.

	**Mother verb–Mother action**		**Mother verb–Child action**	
	**Shared repertoire**	**Total mother repertoire**	**Shared repertoire**	**Total mother repertoire**
Verb type	25	79	24	77
Redundancy %	31.6		31.2	
Verb token	789	1055	631	864
Redundancy %	74.8		73	

*Participants, sessions, and situations are aggregated.*

## Discussion

This study examines the early mother-child multisensory communication by searching for the temporal co-occurrence of verbs and actions in naturalistic scenarios. The results showed that during the daily routines, the mother and child produce manual verbs (2.3 per minute) while they are actively engaged in a continuous stream of linked manual actions (11.2 per minute). This evidence confirms the multidimensional nature of the early communicative and manipulative scenario, full of visual-motor and visual-auditory coordination (Bahrick and Lickliter, 2000). The results also confirmed the expected 1-year delay between the performance of basic manual actions already established at 12 months and the production of manual verbs in children around 24 months (Buresh et al., 2006), whereas mothers produced verbs and actions from the very beginning in their communication with the child. Compared with the child, mothers dominate the verb production (eight times more) while the manual activity was more balanced (1.3 times more), indicating the maternal efforts to fill in the silent gap with a rich milieu of verbs and actions.

An important issue addressed in our study is whether verb-action co-occurrences happened more than at the chance level and within a short time interval, two conditions that should be met in the early multidimensional scenario to help infants to establish efficient patterns for selectively attending to relevant and coherent aspects of their environment (Bahrick and Lickliter, 2000). We found evidence that producing verbs and performing matching actions co-occurred well beyond chance level, since the probability of these matching instances was five to six times higher in each group than the random probability. This is the case even when the probability of random occurrence of verbs and action was calculated in the whole set of instances across the temporal stream whereas the probability of matched co-occurrence was calculated in the subset of matched instances (74%) and time window (±60 s) where a given verb corresponded with the action.

As expected, the temporal distributions of verb-action instances in both groups were quite symmetrical and skewed with the mode around zero seconds, indicating that synchronic instances were the most frequent cases: 8.7 and 7% in the two groups, respectively. Extending the temporal range to ±5 s the number of instances reached 40% in both groups. The verb tends to precede the action more frequently than the action precedes the verb. Compared to the short time (less than half a second) in which 8-month-old infants discriminate between a non-verbal sound and a green disk (Lewkowicz, 2000), noticing audio-visual synchrony with more complex entities require longer time intervals, since the representation of manual verbs and actions extended more in time than that of simple psychophysical stimuli. Convergently, in a task similar to ours, mothers’ verb-action instances were fixed at ±2 s when analyzing them as an input to 6-month-old infants (Nomikou et al., 2017). As in our case, these authors also found that instances in which the action comes after the verb were produced significantly more often than when the action comes before the verb. It seems that “announcing” and “reporting” the action involves a pragmatic function that directs the child’s attention to future and past events, helping to link verbs and actions in a wider representational frame. In sum, the two conditions of inter-sensorial early communication that involve high chances of being exposed to verb-action co-occurrences with a short time interval are met to support more efficiently the verb-referent pairing.

When examining the agents involved in the matched instances, there is evidence that the frequencies of mother-led and child-led categories are both modulated by the age months/group, showing that the interactive game is intertwined with the cross-modal links according to the child’s development and maternal supporting role (Yu and Smith, 2012, 2016). The mother-led categories occurred in both age groups from the very beginning (12 and 24 months) and remain sustained across sessions in parallel with the continuous presence of mothers’ verbs, unlike in the mothers’ mismatching sentences in Group 1 that started later on at 18 months. In turn, the child-led categories were restricted to Group 2 and drastically increased at 30 months where the child’s production of verbs became robust, like in the child’s mismatching instances. Therefore, matching and mismatching instances shared the same developmental constraints but differ in the maternal role being more supportive in the matched instances during the child’s silent period (12–18 months). Comparing the temporal distributions of the matched verb-action instances in the four categories, the majority of instances occurred in a short time interval, with high symmetry and skewness being the rule. The trend in the mother-led instances was that the verb was announcing the action, whereas in the child-led instances this bias disappeared or was reversed, with the verb commenting on the action. This difference may be due to the guiding role of the mother in monitoring the daily routine combined with the greater age-related difficulties in the child’s production of verbs, indexed by the temporal delay after the action performed.

Examining mother-child interactive agency in verb-action co-occurrences offers new insights into the type of exchanges that spontaneously happen in the early communicative scene. The “Mother verb–Mother action” and the “Mother verb–Child action” categories occurred from the very beginning in Group 1 and remained in Group 2. As a result, infants were receiving a continuous and very specific input of matched actions and verbs co-occurring quite synchronously, while they were watching what the mother was doing and listening to what she was saying about her own actions. Another form of maternal support was observed when the child’s perception of the mother’s verbs was contingently associated with the child’s own actions (Goldfield, 2000). Interestingly, both types of maternal support occurred very early, in a time gap in which infants already manipulate objects (before 12 months old) but are still on their way to producing manual verbs (verb onset at 24 months). Infants from 12 months on are able to interpret and react to maternal actions as goal-directed motor activity whether they are included or not in their own repertoire of actions, facilitating their covert reproduction of the observed actions (Cannon et al., 2012; Southgate and Begus, 2013), whereas infants at 13–15 months are also able to categorize events (Konishi et al., 2016). Likewise, infants at 12 months are able to segment verbs in adult speech (Nazzi et al., 2005; Swingley, 2009) and at 21 months they can map perceptually salient actions into words (Brandone et al., 2007). Therefore, it is likely that infants in our sample from 12 months on were able to associate their perception of the mother’s verb with their concurrent encoding of the mother’s action. The child’s ability to connect the observed action and the perceived verb is likely to be crucial in the initial stages of the acquisition of embodied meaning for action verbs (e.g., Glenberg and Gallese, 2012). Given the early preference for multisensory and temporally synchronous input (Gogate and Hollich, 2010), it is also likely that children’s learning may benefit from this maternal verbal input and their own coordinated actions. Recent evidence showed that 6-month-old infants’ exposure to the two mother-led categories predicted their number of spoken verbs at 24 months (Nomikou et al., 2017).

The child-led categories (“Child verb–Child action” and the “Child verb–Mother action”) were only found in Group 2, when the child’s production of manual verbs was robust (403 at a rate of 0.5 per minute), according to Table 2. The first category, involving the child producing a verb while performing a coordinated action, is consistent with what has been called “egocentric speech,” a monolog in which children talk to themselves about what they are doing at that moment (e.g., Piaget, 1923/1955; Vygotsky, 1934/1962). Consistently, an experimental study reported that children (aged 24–26 months) produced 90% of utterances with verbs to describe their own actions (Huttenlocher et al., 1983). With respect to the child describing the mother’s actions, in experimental settings it has been found that children who were taught new action words were able to describe another’s actions when explicitly requested (Childers and Tomasello, 2006); also, a study using the diary method reported others’ actions being referenced by the child when several interlocutors were present (Naigles et al., 2009). The presence of child-led categories limited to Group 2 indicates that these children have already succeeded to some extent in setting up the verb-action links and are able to deal with them either when applied to their own activity or to the mother’s activity. Taken together, the developmentally changing scenes (Smith et al., 2018) provide different learning opportunities, mainly supported by the mother but also led by the child’s initiative and responsivity, to experience types of action-verb pairings that may play a different role during the verb acquisition and consolidation processes.

For the second objective, we explored two pragmatic features of the communicative settings that may facilitate the verb-action pairings: referential alignment and redundancy. To this end, we fist inspected the list of mother-child shared verbs that co-occur with actions and the objects referred to, and we found an alignment of their referential space consisting of the preferred use of general verbs (applicable to a high variety of objects), which may facilitate in both agents a fast mapping with their meaning (Casenhiser and Goldberg, 2005). Mother and child also used more specific verbs with fewer associated objects, but in this case the mother used them more frequently than the child. A previous diary study indicated that children use their newly acquired verbs flexibly in multiple situations with a variety of associated objects, appearing to be learning both general and specific verbs at the same time (Childers, 2009; Naigles et al., 2009). Our study added that the mother plays a differential supportive role by rehearsing and practicing general verbs known by the child, but also by offering typical examples of specific verbs in a variety of instances to facilitate expanding the verb vocabulary (Poulin-Dubois and Forbes, 2006).

Concerning the second pragmatic feature, we found a relatively low mother-child redundancy in the verb types (31%) and a high redundancy in the verb tokens (74%) in the shared repertoire. Corpora studies have emphasized the high co-variation that exists between the mother’s and child’s verb production, but they do not provide any temporal data (Hills, 2013). Our study added that the time-framed convergence in tokens between mother and child may facilitate the rehearsal and practice of known verbs in a great variety of situations, while the maternal low convergence in types opens the venue for learning novel verbs. Both kinds of maternal semantic alignments to the child’s referential space and verb repertoire resemble Vygotsky’s (1934/1962) notion of zone of proximal development, according to which the interaction with adults determines the horizon of what is near to be acquired.

As for limitations, we recognize that the sample is small. Yet, this was compensated by the follow-up at five time points and by the exhaustive temporal recording of instances in both mothers and children. Second, the study of verb-action contingencies was confined to the manipulative domain, which did not exclude the possibility of finding more mismatched instances when including postural verbs and postural actions that may add noise to the input. For this reason we are cautious about the potential distinctiveness of verb-action contingencies in the manual actions with respect to other action domains. Third, our list of mother and child verbs cannot be generalized to other samples, since this is an observational study done with a small sample of mother-child dyads and with a focus on action verbs collected during family daily routines and limited to certain age groups. This approach may have primed some action verbs over others, given that action verbs are contextually dependent (Childers and Tomasello, 2006). However, supporting the appropriateness of the sampling is the finding that about half of the children’s manipulative verbs are contained in the list of children’s earlier English verbs identified by Tomasello (1992), and all of them appeared as early acquired verbs in adult normative studies in Spanish (Cuetos and Alija, 2003; Alonso et al., 2016). Finally, we did not include independent measures of child’s locomotor and manipulative skills, which could be a significant predictor of the verb vocabulary production in the range of ages studied.

## Conclusion

In conclusion, the adoption of a naturalistic bottom-up approach has provided new developmental evidence showing that early mother-child communication in the manipulative scenario is well prepared to facilitate the verb-referent pairings. This facilitation is orchestrated by the presence of time-framed action-verb associations, involving systematically the child, the mother or both, operating over the second and third years of life. These findings help to delineate four emerging properties of verb-action contingencies that may facilitate the referential grounding of manual verbs on the ongoing manipulative actions: (1) *Cross-modal and temporal adjustment*: Verb-action co-occurrences, far beyond the chance level and within a short time interval, do exist in the child’s communicative environment from a very early age. (2) *Variety of sources*: Verb-action patterns involve one agent (the child or the mother) and two agents (the child and the mother), providing the support of social interactive sources. (3) *Accuracy*: Verb-action contingencies showed a referential matching, providing reliable on-line connections between specific manual verbs and the corresponding actions. Finally, (4) *Referential alignment and redundancy:* Mothers aligned their verbs’ referential space to that of the child’s verbs, and mother-child verbs greatly overlapped within time-framed instances, far beyond a mere linguistic covariance. In conclusion, the early multisensory communicative scene affords grounding of verb meanings on the ongoing actions, facilitating the action-verb pairing in the realm of social interactions despite the prevailing solipsistic approach to embodiment.

## Data Availability Statement

The raw data supporting the conclusions of this article will be made available by the authors, without undue reservation.

## Ethics Statement

This study was carried out in accordance with the recommendations of the Ethics Committee of the University of la Laguna, Spain. In accordance with the Declaration of Helsinki, a written informed consent was obtained from the mothers. Written informed consent to participate in this study was provided by the participants’ legal guardian/next of kin.

## Author Contributions

MR made a contribution to the study design, data analysis and interpretation, and the manuscript drafting and revising. MM-A participated in collecting data, coding and analysis, interpretation of the data, as well as the manuscript revising. MV made a contribution to the study design, interpretation of data, the manuscript drafting and revising. All authors have read and approved the final manuscript.

## Conflict of Interest

The authors declare that the research was conducted in the absence of any commercial or financial relationships that could be construed as a potential conflict of interest.

## References

[B1] Alonso M. Á. Díez E. Fernández A. (2016). Subjective age-of-acquisition norms for 4,640 verbs in Spanish. *Behav. Res. Methods* 48 1337–1342. 10.3758/s13428-015-0675-z 26494650

[B2] Antognini K. Daum M. M. (2019). Toddlers show sensorimotor activity during auditory verb processing. *Neuropsychologia* 126 82–91. 10.1016/j.neuropsychologia.2017.07.022 28734698

[B3] Bahrick L. E. Lickliter R. (2000). Intersensory redundancy guides attentional selectivity and perceptual learning in infancy. *Dev. Psychol.* 36 190–201. 10.1037/0012-1649.36.2.190 10749076 PMC2704001

[B4] Barsalou L. W. (1999). Perceptual symbol system. *Behav. Brain Sci.* 22 577–660. 10.1017/S0140525X99002149 11301525

[B5] Behne T. Carpenter M. Call J. Tomasello M. (2005). Unwilling versus unable: infants’ understanding of intentional action. *Dev. Psychol.* 41 328–337. 10.1037/0012-1649.41.2.328 15769189

[B6] Boulenger V. Roy A. C. Paulignan Y. Deprez V. Jeannerod M. Nazir T. A. (2006). Cross-talk between language processes and overt motor behavior in the first 200 ms of processing. *J. Cogn. Neurosci.* 18 1607–1615. 10.1162/jocn.2006.18.10.1607 17014366

[B7] Brandone A. C. Pence K. L. Golinkoff R. M. Hirsh-Pasek K. (2007). Action speaks louder than words: young children differentially weight perceptual, social, and linguistic cues to learn verbs. *Child Dev.* 78 1322–1342. 10.1111/j.1467-8624.2007.01068.x 17650141

[B8] Buccino G. Riggio L. Melli G. Binkofski F. Gallese V. Rizzolatti G. (2005). Listening to action-related sentences modulates the activity of the motor system: a combined TMS and behavioral study. *Cogn. Brain Res.* 24 355–363. 10.1016/j.cogbrainres.2005.02.020 16099349

[B9] Buresh J. S. Woodward A. Brune C. W. (2006). “The roots of verbs in prelinguistic action knowledge,” in *Action Meets Word: How Children Learn Verbs*, eds Hirsh-Paske K. Golinkoff R. M. (New York, NY: Oxford University Press), 208–227. 10.1093/acprof:oso/9780195170009.003.0009

[B10] Cannon E. N. Woodward A. L. Gredebäck G. Von Hofsten C. Turek C. (2012). Action production influences 12-month-old infants’ attention to others’ actions. *Dev. Sci.* 15 35–42. 10.1111/j.1467-7687.2011.01095.x 22251290 PMC3261504

[B11] Casenhiser D. Goldberg A. E. (2005). Fast mapping between a phrasal form and meaning. *Dev. Sci.* 8 500–508. 10.1111/j.1467-7687.2005.00441.x 16246241

[B12] Childers J. B. (2009). Early verb learners: creative or not? In L. R. Naigles, E. Hoff & D. Vear, Flexibility in early verb use: evidence from a multiple-N diary study. *Monogr. Soc. Res. Child Dev.* 74 133–139. 10.1111/j.1540-5834.2009.00524.x 19660058

[B13] Childers J. B. Tomasello M. (2006). “Are nouns easier to learn than verbs? Three experimental studies,” in *Action Meets Word: How Children Learn Verbs*, eds Hirsh-Pasek K. Golinkoff R. M. (New York, NY: Oxford University Press), 311–335. 10.1093/acprof:oso/9780195170009.003.0013

[B14] Cuetos F. Alija M. (2003). Normative data and naming times for action pictures. *Behav. Res. Methods Instrum. Comput.* 35 168–177. 10.3758/bf03195508 12723791

[B15] de Vega M. (2008). “Levels of embodied meaning. From pointing to counterfactuals,” in *Symbols and Embodiment: Debates on Meaning and Cognition*, eds de Vega M. Glenberg A. Graesser A. (New York, NY: Oxford University Press), 285–308. 10.1093/acprof:oso/9780199217274.003.0014

[B16] Gampe A. Daum M. M. (2014). Productive verbs facilitate action prediction in toddlers. *Infancy* 19 301–325. 10.1111/infa.12047

[B17] García A. M. Ibáñez A. (2018). When embodiment breaks down: language deficits as novel avenues into movement disorders. *Cortex* 100 1–7. 10.1016/j.cortex.2017.12.022 29496261

[B18] Gentner D. (2006). “Why verbs are hard to learn,” in *Action Meets Word: How Children Learn Verbs*, eds Hirsh-Paske K. Golinkoff R. M. (New York, NY: Oxford University Press), 544–564. 10.1093/acprof:oso/9780195170009.003.0022

[B19] Gleitman L. R. Cassidy K. Nappa R. Papafragou A. Trueswell J. C. (2005). Hard Words. *Lang. Learn. Dev.* 1 23–64. 10.1207/s15473341lld0101_4

[B20] Glenberg A. M. (1997). What memory is for. *Behav. Brain Sci.* 20 1–19; discussion 19–55. 10.1017/S0140525X97000010 10096994

[B21] Glenberg A. M. Gallese V. (2012). Action-based language: a theory of language acquisition, comprehension, and production. *Cortex* 48 905–922. 10.1016/j.cortex.2011.04.010 21601842

[B22] Gogate L. J. Hollich G. (2010). Invariance detection within an interactive system: a perceptual gateway to language development. *Psychol. Rev.* 117 496–516. 10.1037/a0019049 20438235

[B23] Goldfield B. A. (2000). Nouns before verbs in comprehension vs. production: the view from pragmatics. *J. Child Lang.* 27 501–520. 10.1017/S0305000900004244 11089337

[B24] Herrera E. Cuetos F. (2012). Action naming in Parkinson’s disease patients on/off dopamine. *Neurosci. Lett.* 513 219–222. 10.1016/j.neulet.2012.02.045 22387157

[B25] Hills T. (2013). The company that words keep: comparing the statistical structure of child- versus adult-directed language. *J. Child Lang.* 40 586–604. 10.1017/S0305000912000165 22584041

[B26] Hirsh-Pasek K. Golinkoff R. M. (eds). (2006). *Action Meets Word: How Children Learn Verbs.* New York, NY: Oxford University Press.

[B27] Hollich G. Hirsh-Pasek K. Golinkoff R. M. (2000). What does it take to learn a word? *Monogr. Soc. Res. Child Dev.* 65 1–16. 12467096

[B28] Huttenlocher J. Smiley P. Charney R. (1983). The emergence of action categories in the child: evidence from verb meaning. *Psychol. Rev.* 90 72–93. 10.1037/0033-295X.90.1.72

[B29] Iverson J. M. (2010). Developing language in a developing body: the relationship between motor development and language development. *J. Child Lang.* 37 229–261. 10.1017/S0305000909990432 20096145 PMC2833284

[B30] James K. H. Maouene J. (2009). Auditory verb perception recruits motor systems in the developing brain: an fMRI investigation. *Dev. Sci.* 12 F26–F34. 19840036 10.1111/j.1467-7687.2009.00919.x

[B31] James K. H. Swain S. N. (2011). Only self-generated actions create sensori-motor systems in the developing brain. *Dev. Sci.* 14 673–678. 10.1111/j.1467-7687.2010.01011.x 21676088 PMC4176697

[B32] Katerelos M. Poulin-Dubois D. Oshima-Takane Y. (2011). A cross-linguistic study of word-mapping in 18-to 20-month-old infants. *Infancy* 16 508–534. 10.1111/j.1532-7078.2010.00064.x 32693550

[B33] Klepp A. Van Dijk H. Niccolai V. Schnitzler A. Biermann-Ruben K. (2019). Action verb processing specifically modulates motor behaviour and sensorimotor neuronal oscillations. *Sci. Rep.* 9:15985. 10.1038/s41598-019-52426-9 31690784 PMC6831701

[B34] Konishi H. Stahl A. E. Golinkoff R. M. Hirsh-Pasek K. (2016). Individual differences in nonlinguistic event categorization predict later motion verb comprehension. *J. Exp. Child Psychol.* 151 18–32. 10.1016/j.jecp.2016.03.012 27139436

[B35] Lewkowicz D. J. (2000). The development of intersensory temporal perception: an epigenetic systems/limitations view. *Psychol. Bull.* 126 281–308. 10.1037/0033-2909.126.2.281 10748644

[B36] Lifter K. Bloom L. (1989). Object knowledge and the emergence of language. *Infant Behav. Dev.* 12 395–423. 10.1016/0163-6383(89)90023-4

[B37] Loeffler J. Raab M. Cañal-Bruland R. (2016). A lifespan perspective on embodied cognition. *Front. Psychol.* 7:845. 10.3389/fpsyg.2016.00845 27313562 PMC4887461

[B38] Maouene J. Laakso A. Smith L. B. (2011). Object associations of early-learned light and heavy English verbs. *First Lang.* 31 109–132. 10.1177/0142723710380528 24363471 PMC3867984

[B39] Moreno I. de Vega M. León I. Bastiaansen M. Glen Lewis A. Magyari L. (2015). Brain dynamics in the comprehension of action-related language. A time-frequency analysis of mu rhythms. *Neuroimage* 109 50–62. 10.1016/j.neuroimage.2015.01.018 25583610

[B40] Naigles L. R. Hoff E. Vear D. (2009). Flexibility in early verb use: evidence from a multiple-n diary study. *Monogr. Soc. Res. Child Dev.* 74 vii–112. 10.1111/j.1540-5834.2009.00513.x 19660058

[B41] Nazzi T. Dilley L. C. Jusczyk A. M. Shattuck-Hufnagel S. Jusczyk P. (2005). English-learning infants’ segmentation of verbs from fluent speech. *Lang. Speech* 48 279–298. 10.1177/00238309050480030201 16416938

[B42] Nomikou I. Koke M. Rohlfing K. J. (2017). Verbs in mothers’ input to six-month-olds: synchrony between presentation, meaning, and actions is related to later verb acquisition. *Brain Sci.* 7:52. 10.3390/brainsci7050052 28468265 PMC5447934

[B43] Oliveri M. Finocchiaro C. Shapiro K. Gangitano M. Caramazza A. Pascual-Leone A. (2004). All talk and no action: a transcranial magnetic stimulation study of motor cortex activation during action word production. *J. Cogn. Neurosci.* 16 374–381. 10.1162/089892904322926719 15072673

[B44] Oudgenoeg-Paz O. Volman M. C. Leseman P. P. (2016). First steps into language? Examining the specific longitudinal relations between walking, exploration and linguistic skills. *Front. Psychol.* 7:1458. 10.3389/fpsyg.2016.01458 27729885 PMC5037183

[B45] Papeo L. Vallesi A. Isaja A. Rumiati R. I. (2009). Effects of TMS on different stages of motor and non-motor verb processing in the primary motor cortex. *PLoS One* 4:e4508. 10.1371/journal.pone.0004508 19240793 PMC2643000

[B46] Piaget J. (1923/1955). *The Language and Thought of the Child.* New York, NY: Meridian.

[B47] Poulin-Dubois D. Forbes J. N. (2006). “Word, intention, and action: a two-tiered model of action word learning,” in *Action Meets Word: How Children Learn Verbs*, eds Hirsh-Pasek K. Golinkoff R. M. (New York, NY: Oxford University Press), 262–285. 10.1093/acprof:oso/9780195170009.003.0011

[B48] Pulvermüller F. (1999). Words in the brain’s language. *Behav. Brain Sci.* 22 253–279. 11301524

[B49] Pulvermüller F. (2005). Brain mechanisms linking language and action. *Nat. Rev. Neurosci.* 6 576–582. 10.1038/nrn1706 15959465

[B50] Pulvermüller F. (2008). “Grounding language in the brain,” in *Symbols, Embodiment, and Meaning*, eds de Vega M. Graesser A. Glenberg A. M. (Oxford: Oxford University Press), 85–116. 10.1093/acprof:oso/9780199217274.003.0006

[B51] Reynolds G. D. Bahrick L. E. Lickliter R. Guy M. W. (2014). Neural correlates of intersensory processing in 5-month-old infants. *Dev. Psychobiol.* 56 355–372. 10.1002/dev.21104 23423948 PMC3954462

[B52] Smith L. B. Jayaraman S. Clerkin E. Yu C. (2018). The developing infant creates a curriculum for statistical learning. *Trends Cogn. Sci.* 22 325–336. 10.1016/j.tics.2018.02.004 29519675 PMC5866780

[B53] Southgate V. Begus K. (2013). Motor activation during the prediction of nonexecutable actions in infants. *Psychol. Sci.* 24 828–835. 10.1177/0956797612459766 23678509 PMC3938142

[B54] Swingley D. (2009). Contributions of infant word learning to language development. *Philos. Trans. R. Soc. B Biol. Sci.* 364 3617–3632. 10.1098/rstb.2009.0107 19933136 PMC2828984

[B55] Tamis-LeMonda C. S. Kuchirko Y. Luo R. Escobar K. Bornstein M. H. (2017). Power in methods: language to infants in structured and naturalistic contexts. *Dev. Sci.* 20:e12456. 10.1111/desc.12456 28093889 PMC5865594

[B56] Tomasello M. (1992). *First Verbs: A Case Study of Early Grammatical Development.* Cambridge: Cambridge University Press.

[B57] van Elk M. van Schie H. T. Zwaan R. A. Bekkering H. (2010). The functional role of motor activation in language processing: motor cortical oscillations support lexical-semantic retrieval. *Neuroimage* 50 665–677. 10.1016/j.neuroimage.2009.12.123 20060478

[B58] Vygotsky L. S. (1934/1962). *Thought and language.* Cambridge, MA: MIT Press.

[B59] Walle E. A. Campos J. J. (2014). Infant language development is related to the acquisition of walking. *Dev. Psychol.* 50 336–348. 10.1037/a0033238 23750505

[B60] Waxman S. R. Lidz J. L. (2006). “Early word learning,” in *Handbook of Child Psychology: Cognition, Perception and Language*, eds Kuhn D. Siegler R. (New York, NY: Wiley), 299–335.

[B61] Wellsby M. Pexman P. M. (2014). Developing embodied cognition: insights from children’s concepts and language processing. *Front. Psychol.* 5:506. 10.3389/fpsyg.2014.00506 24904513 PMC4036138

[B62] Yu C. Smith L. B. (2012). Embodied attention and word learning by toddlers. *Cognition* 125 244–262. 10.1016/j.cognition.2012.06.016 22878116 PMC3829203

[B63] Yu C. Smith L. B. (2016). The social origins of sustained attention in one-year-old human infants. *Curr. Biol.* 26 1235–1240. 10.1016/j.cub.2016.03.026 27133869 PMC5387765

[B64] Zwaan R. A. (2008). “Experiential traces and mental simulations in language comprehension,” in *Symbols, Embodiment, and Meaning*, eds De Vega M. Glenberg A. M. Graesser A. C. (Oxford: Oxford University Press), 165–180. 10.1093/acprof:oso/9780199217274.003.0009

