# Corrigendum: Exploring the Co-occurrence of Manual Verbs and Actions in Early Mother-Child Communication

**DOI:** 10.3389/fpsyg.2020.628409

**Published:** 2020-12-04

**Authors:** María José Rodrigo, Mercedes Muñetón-Ayala, Manuel de Vega

**Affiliations:** ^1^Facultad de Psicología, Universidad de La Laguna, San Cristóbal de La Laguna, Spain; ^2^Facultad de Comunicaciones, Universidad de Antioquia, Medellín, Colombia; ^3^Instituto Universitario de Neurociencias, Universidad de La Laguna, San Cristóbal de La Laguna, Spain

**Keywords:** verb-action co-occurrence, temporal synchrony, social interaction, embodied meaning, multisensory communication

In the published article, there were errors in affiliations 1 and 2. For affiliation 1, instead of “Facultad de Comunicación, Universidad de Antioquia, Medellín, Colombia” it should be “Facultad de Psicología, Universidad de La Laguna, San Cristóbal de La Laguna, Spain”. For affiliation 2, instead of “Facultad de Psicología, Universidad de La Laguna, San Cristóbal de La Laguna, Spain” it should be “Facultad de Comunicaciones, Universidad de Antioquia, Medellín, Colombia.”

Additionally, the author list displayed the wrong affiliation numbers for authors Mercedes Muñetón-Ayala and Manuel de Vega. Instead of “María José Rodrigo^1^^*^, Mercedes Muñetón-Ayala^2, 3^ and Manuel de Vega^2, 3^,” it should be “María José Rodrigo^1^^*^, Mercedes Muñetón-Ayala^2^ and Manuel de Vega^3^.”

The authors apologize for these errors and state that they do not change the scientific conclusions of the article in any way. The original article has been updated.

