# Supplementary material for: Exploring the Co-occurrence of Manual Verbs and Actions in Early Mother-Child Communication
Source: Front Psychol. 2020 Nov 10;11:596080. doi: 10.3389/fpsyg.2020.596080 (PMC7683411; doi:10.3389/fpsyg.2020.596080)
Supplement: Supplementary file 1 [file Data_Sheet_1.PDF]

Supplementary Material

Supplementary Table

Table 1S. Frequency (Vf) and percent (%) of the verb repertoire shared by the mother and child, number of objects referred to (NObj) and by verb frequency (NObj/Vf), produced in the child-led and mother-led categories, collapsed across age groups.

Verbs		Mother verb-Mother action			Mother verb-Child action			Child verb-Child action			Child verb-Mother action		
**Spanish**	**English**	**Vf (%)**	**NObj**	***NObj/Vf***	**Vf (%)**	**NObj**	***NObj/Vf***	**Vf (%)**	**NObj**	***NObj/Vf***	**Vf (%)**	**NObj**	***NObj/Vf***
tirar	throw	21 (2.66)	12	0.57	65 (10.30)	17	0.26	34 (27.20)	8	0.24	3 (3.95)	3	1.00
poner	put	227 (28.77)	64	0.28	115 (18.23)	33	0.29	9 (7.20)	4	0.44	14 (18.42)	7	0.50
coger	catch	102 (12.93)	26	0.25	81 (12.84)	26	0.32	9 (7.20)	8	0.89	3 (3.95)	3	1.00
dar	give	72 (9.13)	28	0.39	76 (12.04)	24	0.32	9 (7.20)	6	0.67	7 (9.21)	2	0.29
romper	break	3 (0.38)	3	1.00	14 (2.22)	11	0.79	8 (6.40)	2	0.25	0 (0)	0	0
lavar	wash	35 (4.44)	9	0.26	39 (6.18)	13	0.33	7 (5.60)	4	0.57	7 (9.21)	4	0.57
pintar	paint	17 (2.15)	5	0.29	26 (4.12)	6	0.23	6 (4.80)	3	0.50	2 (2.63)	2	1.00
quitar	remove	70 (8.87)	27	0.39	37 (5,86)	17	0.46	6 (4.80)	4	0.67	17 (22.37)	10	0.59
pegar	paste	6 (0.76)	3	0.50	1 (0.16)	1	1.00	4 (3.20)	2	0.50	1 (1.32)	1	1.00
peinar	comb	11 (1.39)	2	0.18	32 (5.07)	5	0.16	4 (3.20)	3	0.75	2 (2.63)	1	0.50
abrochar	fasten	4 (0.51)	3	0.75	3 (0.48)	1	0.33	3 (2.40)	1	0.33	1 (1.32)	1	1.00
cortar	cut	3 (0.38)	2	0.67	7 (1.11)	5	0.71	3 (2.40)	2	0.67	1 (1.32)	1	1.00
recoger	pick up	9 (1.14)	4	0.44	8 (1.27)	5	0.63	3 (2.40)	3	1.00	3 (3.95)	2	0.67
tomar	take	79 (10.01)	29	0.37	32 (5.07)	16	0.50	3 (2.40)	3	1.00	3 (3.95)	3	1.00
hacer	make	27 (3.42)	18	0.67	15 (2.38)	12	0.80	2 (1.60)	2	1.00	2 (2.63)	2	1.00
abrir	open	15 (1.9)	8	0.53	11 (1.74)	7	0.64	2 (1.60)	2	1.00	1 (1.32)	1	1.00
buscar	look	14 (1.77)	5	0.36	9 (1.43)	3	0.33	2 (1.60)	1	0.50	0 (0)	0	0
sobar	knead	0 (0)	0	0	0 (0)	0	0	2 (1.60)	1	0.50	0 (0)	0	0
echar	pour	42 (5.32)	9	0.21	19 (3.01)	8	0.42	1 (0.80)	1	1.00	1 (1.32)	1	1.00
llevar	carry	10 (1.27)	4	0.40	10 (1.58)	5	0.50	1 (0.80)	1	1.00	1 (1.32)	1	1.00

Verbs		Mother verb-Mother action			Mother verb-Child action			Child verb-Child action			Child verb-Mother action		
**Spanish**	**English**	**Vf (%)**	**NObj**	***NObj/Vf***	**Vf (%)**	**NObj**	***NObj/Vf***	**Vf (%)**	**NObj**	***NObj/Vf***	**Vf (%)**	**NObj**	***NObj/Vf***
apretar	tighten	1 (0.13)	1	1.00	1 (0.16)	1	1.00	1 (0.80)	1	1.00	0 (0)	0	0
arreglar	fix	1 (0.13)	1	1.00	4 (0.63)	3	0.75	1 (0.80)	1	1.00	1 (1.32)	1	1.00
encestar	dunk	1 (0.13)	1	1.00	0 (0)	0	0	1 (0.80)	1	1.00	0 (0)	0	0
enjuagar	rinse	0 (0)	0	0	0 (0)	0	0	1 (0.80)	1	1.00	0 (0)	0	0
mojar	wet	13 (1.65)	4	0.31	2 (0.32)	2	1.00	1 (0.80)	1	1.00	4 (5.26)	2	0.50
tener	have	4 (0.51)	3	0.75	6 (0.95)	4	0.67	1 (0.80)	1	1.00	1 (1.32)	1	1.00
traer	bring	2 (0.25)	2	1.00	18 (2.85)	11	0.61	1 (0.80)	1	1.00	1 (1.32)	1	1.00
**Total**	**27**	**789**	**273**	**0.35**	**631**	**236**	**0.37**	**125**	**68**	**0.54**	**76**	**50**	**0.66**
